# The Chinese version of the cardiac depression scale: Mokken scaling

**DOI:** 10.1186/1477-7525-10-33

**Published:** 2012-03-27

**Authors:** Roger Watson, Wenru Wang, David L Hare, Chantal F Ski, David R Thompson

**Affiliations:** 1 School of Nursing& Midwifery, University of Hull, Hull, UK; 2 Alice Lee Centre for Nursing Studies, National University of Singapore, Singapore; 3 Department of Medicine, University of Melbourne, Melbourne, Australia; 4 Cardiovascular Research Centre, Australian Catholic University, Melbourne, Australia

**Keywords:** Cardiac Depression Scale, Depression, Psychometrics, Mokken scaling

## Abstract

**Background:**

Myocardial infarction is a major cause of death and morbidity in many countries, including China. The aim of this study was to analyse a Mandarin Chinese translation of the Cardiac Depression Scale for a hierarchy of items according to the criteria of Mokken scaling.

**Findings:**

Data from 438 Chinese participants who completed the Chinese translation of the Cardiac Depression Scale were analysed using the Mokken scaling procedure and the 'R' statistical programme using the diagnostics available in these programmes. Correlations between Mandarin Chinese items and Chinese translations of the Hospital Anxiety and Depression Scale and the Beck Depression Inventory were also analysed. Fifteen items from the Mandarin Chinese Cardiac Depression Scale were retained in a weak but reliable Mokken scale; invariant item ordering was evident but of low accuracy and the Mokken scaled items of the Chinese Cardiac Depression Scale correlated with the Hospital Anxiety and Depression Scale and the Beck Depression Inventory.

**Conclusions:**

Items from the Mandarin Chinese Cardiac Depression Scale form a Mokken scale and this offers further insight into how the items of the Cardiac Depression Scale relate to the measurement of depression in people with a myocardial infarction.

## Background

It is well recognised that myocardial infarction (MI) remains a major cause of death and morbidity in the West and has profound consequences on health-related quality of life (HRQoL) and this is strongly related to the depression experienced by people who have had an MI. A scale specific to the measurement of depression in MI, the Cardiac Depression Scale (CDS), has been developed [1] to overcome the lack of specificity and responsiveness to change over time of existing measures of depression so that this can be measured more accurately in people with MI. MI is also, increasingly, a major cause of death and morbidity in China and the CDS has been translated and validated in Chinese Mandarin to form the Ch-CDS [2] and this has been validated against exiting measures of depression, the Hospital Anxiety and Depression Scale (HADS) and the Beck Depression Inventory (BDI). However, the CDS and the Ch-CDS have 25 items and there is merit in searching for shorter forms of the scale - for ease of use and administration in clinical practice - and Mokken scaling is one method, recently applied to a range of psychological instruments, whereby this can be achieved [3-8].

## Methods

This study was designed to analyse the Ch-CDS using exploratory Mokken scaling [9] to investigate if there was a unidimensional hierarchical scale in data gathered in China and to investigate the concurrent validity of the Ch-CDS against validated Chinese Mandarin versions of the HADS and BDI. Therefore, this was an empirically, as opposed to theoretically, driven study. This was a secondary analysis of two dataset gathered from Chinese MI patients (n = 438) who completed the Ch-CDS. Ethical permission was obtained from the university and hospital in Xi'an, China where the study was conducted. All participants gave written informed consent. The demographics of the sample were: males (n = 286); females (n = 158); mean age 56 years (range 18-87, SD = 15). One dataset (n = 200) included the Ch-HADS [2] and the other (n = 238) included the Ch-BDI [10].

The Ch-CDS is a 25-item scale with a seven option Likert-type response format from 'Strongly agree to 'Strongly disagree'. Data, with reverse scored items re-coded, were entered into Statistical Package for the Social Sciences (SPSS) version 16.0 and imported into the Mokken scaling analysis for polytomous items (MSP) software [11]. MSP is a computer programme that searches polytomous data for hierarchical scales using a range of diagnostic criteria. Data were also imported into the 'R' programme version 2.11.1 and, using the Mokken scaling analysis procedure in 'R', and analysed for invariant item ordering (IIO), to be considered below. SPSS was used to analyse Spearman's rho between the Ch-CDS data and the Ch-HADS and Ch-BDI, separately.

As previously explained, Mokken scaling is a method related to item response theory and recent publications [3-8] have explained the nature of Mokken scales in non-technical language and also applications of Mokken scaling to HRQoL, activities of daily living and psychological interventions. Mokken scaling was used in the present study as opposed to other item-response models, for example Rasch modelling. Theoretical and empirical comparison of Mokken and Rasch models of item response theory [12] show that in situations where all this is required is the ordering of people on a latent trait that Mokken scaling, being less restrictive than Rasch modelling, is sufficient and guarantees ordinal measurement of persons on the latent trait, provided the model applies.

The diagnostics required to evaluate Mokken scaling analyses include Loevinger's coefficient (H) which is used to select items from larger items sets into undimensional item clusters; H > 0.3 indicates weak scale with H > 0.4 indicating a medium scale and H > 0.5 a strong scale with higher values of H indicating greater accuracy in person ordering. The reliability of Mokken scales is estimated using Rho which is a test-retest reliability coefficient with Rho > 0.7 considered to indicate a reliable scale [9]. A Bonferroni correction is used to control for Type 1 error rate in testing whether H is positive during the item selection procedure. Monotone homogeneity - an increase in the score on an item as the latent trait increases - can also be estimated using the 'Crit' value - a value generated by the MSP based on a range of criteria [9] - which estimates violations of monotone homogeneity and double monotonicity. The MSP was run using the default settings of H > 0.3 and *p *< 0.05.

IIO exists where the order of items in a scale, ordered by mean item scores in a group of respondents, is the same at all levels of the latent trait being measured [13]. The ability to test polytomous scales for IIO has only recently been possible with the advent of the 'R' programme [14]. The Mokken scaling analysis in R investigates whether item response functions intersect and Htrans (denoted H^T^) is used to investigate the accuracy of the ordering of the resulting item set [15]; values > 0.3 are considered acceptable [16].

## Results

The results of the Mokken scaling analysis are shown in Table 1. Fifteen Ch-CDS items were retained in a weak Mokken scale which was statistically significant and reliable (H = 0.36; Rho = 0.88; *p *= 0.00026). The ordering of items from the most readily to the least readily endorsed runs from 'My memory is (not) as good as it should be' through lack of pleasure and getting less done worrying about dying and there being 'only misery in the future for me'. These 15 items showed IIO but this was at a very low level of accuracy (H^T ^= 0.11). Pearson's correlation between the Ch-CDS scores on the Mokken scaled items and the Ch-HADS were -0.60 and -0.65 for the Anxiety scale and the Depression scale, respectively (*p *< 0.001) and with the Ch-BDI was 0.75 (*p *< 0.001). Taking the direction of scoring for both these instruments into account, these correlations indicate that the greater the level of depression rated using the Ch-CDS the greater the level of anxiety and depression rated using the Ch-HADS and the Ch-BDI.

**Table 1:** Mokken scaling of the Chinese Cardiac Depression Scale with items showing invariant item ordering (n = 438)

Item	Label	Mean	H
14	There is only misery in the future for me	2.52	0.40

13	The possibility of sudden death worries me	2.75	0.37

25	I feel frustrated	2.85	0.45

8	I am not the person I used to be	2.89	0.41

10	I feel like I'm living on borrowed time	2.97	0.35

6	I may not recover completely	3.13	0.37

23	I feel independent and in control of my life	3.15	0.30

19	I gain just as much pleasure from my leisure activities as I used to*	3.21	0.35

16	I get hardly anything done	3.32	0.37

4	I get pleasure from life at present*	3.43	0.38

12	I feel good in spirits*	3.52	0.35

9	I wake up in the early hours of the morning and cannot get back to sleep	3.67	0.32

3	I can't be bothered doing anything much	3.78	0.31

17	My problems are not over yet	3.79	0.35

20	My memory is as good as it always was*	3.85	0.32

H = 0.36; Rho = 0.88; *p *= 0.00026; H^T ^= 0.11; * reverse scored items

## Discussion

The present study has established that there is a unidimensional hierarchical arrangement of a sub-set of items from the Ch-CDS and that this scale shows concurrent validity when correlated with two separate measures of depression and anxiety. The scale has face validity in the sense that items with descriptors such as 'my problems are not over yet', 'I (don't) feel in good spirits' and 'I get hardly anything done', which are readily endorsed, represent less psychological distress than, items such as 'I feel like I'm living on borrowed time', 'the possibility of sudden death worries me' and 'there is only misery in the future for me'. The value of Mokken scaling has been demonstrated in that it relates the score on the Ch-CDS not only to the overall level of the latent trait but to a specific descriptor of the level of distress being experienced. This is analogous to previous work on Mokken scaling of the General Health Questionnaire 30 item version (GHQ-30) [7] where items demonstrating less psychological distress, for example 'been (un)able to face up to your problems' were more readily endorsed than items such as 'felt that life isn't worth living'.

The Mokken scale derived from the Ch-CDS showed IIO but at a very low level of accuracy. This does not obviate the utility of the Mokken scale; it remains a useful instrument for ordering individuals on the basis of their total mean scale score. However, lack of accurate IIO means that the items are not, necessarily responded to in the same order across all levels of the latent trait by all respondents. Investigation of IIO for polytomous items-such as those in the Ch-CDS-is in its infancy with very few publications demonstrating this analysis. Ligtvoet et al [13] only recently published a method for investigating IIO in polytmous items and this was published in parallel with Emons et al's [9] paper demonstrating IIO in only one cluster of five items of the Hospital Anxiety and Depression Scale (HADS). Emons et al [9] concluded that 'firm conclusions about the hierarchy of items is not justified' (page 10). A more recent Mokken scaling analysis of the HADS [17] came to the same conclusion; however, this does not obviate the use of this well established scale, the lack of accuracy in IIO merely indicates that the relationship between the items in the scale and the latent trait is not fully explained.

The Ch-CDS Mokken scale derived here is useful but further development is possible to select items that show IIO and to confirm this structure using Mokken scaling [9]. The Mokken scale derived here is shorter than the original; the selection of items that conform to IIO would shorten it further and increase the utility of the Ch-CDS further. The 25 item Ch-CDS generally takes less than 5 minutes to complete, completing 10 fewer items would reduce this by approximately 2 minutes. Any time saving in a busy clinical setting is clearly an advantage. However, before recommending the routine use of the 15 item Ch-CDS rather than 25 item Ch-CDS for general use, one would need to confirm the shorter scale's accuracy in screening for depression and responsiveness to change in clinical settings. This would best be accomplished using an independent sample.

## Conclusions

A reliable hierarchy of 15 items within the 25-item Ch-CDS has been identified and this shorter scale has face validity and shows concurrent validity with two established measures of psychological distress. The scale is likely to have clinical utility and could be further developed by seeking items with IIO.

## Abbreviations

BDI: Beck Depression Inventory; CDS: Cardiac Depression Scale; Ch-CDS: Mandarin Chinese translation of the CDS; GHQ-30: General Health Questionnaire 30 item version; HADS: Hospital Anxiety and Depression Scale; HRQoL: Health-related quality of life; IIO: Invariant item ordering; MI: Myocardial infarction; MSP: Mokken scaling analysis for polytomous items; SPSS: Statistical package for the social sciences

## Competing interests

The authors declare that they have no competing interests.

## Authors' contributions

RW conducted the Mokken scaling analysis and drafted the manuscript. WW participated in the design of the study and collected the data. DH provided expertise on the CDS and helped to draft the manuscript. CS participated in the design of the study and helped to draft the manuscript. DT conceived the study and participated in the design and coordination and helped to draft the manuscript. All authors read and approved the final manuscript.
